# Echovirus serotype 11 induced sepsis in a young female patient with multiple sclerosis treated with anti-CD20 monoclonal antibody ocrelizumab

**DOI:** 10.1007/s15010-025-02479-y

**Published:** 2025-02-05

**Authors:** A. D. Starosta, J. Ehler, B. Löffler, A. Tannapfel, A. Zipprich, P. A. Reuken, A. Stallmach

**Affiliations:** 1 Department of Internal Medicine III (Nephrology, Rheumatology, Endocrinology), Jena University Hospital - Friedrich Schiller University, Jena, Germany; 2 Department of Anesthesiology and Intensive Care Medicine, Jena University Hospital, Friedrich-Schiller-University, Jena, Germany; 3 Institute of Medical Microbiology, Jena University Hospital - Friedrich Schiller University, Jena, Germany; 4 Institute of Pathology, Ruhr University Bochum, Bochum, Germany; 5 Department of Internal Medicine IV (Gastroenterology, Hepatology and Infectious Diseases), Jena University Hospital - Friedrich Schiller University, Am Klinikum 1, 07743 Jena, Germany

**Keywords:** Anti-CD20-therapy, Echovirus, Enterovirus, Multiple sclerosis, Immunosuppresion, Hepatitis

## Abstract

**Background:**

Enterovirus infection has been described as a cause of severe viral sepsis in humorally immunosuppressed patients.

**Case presentation:**

A 20-year-old female with a history of multiple sclerosis on ocrelizumab therapy with persistent agammaglobulinemia and autoimmune hepatitis treated with azathioprine/budesonide presented with subacute sensorineural hearing loss, hepatitis, pneumonia, enterocolitis and pancreatitis. Molecular pathological techniques detected enterovirus RNA in samples from the liver, blood, ascites fluid, and pleural effusions, confirming Echovirus serotype 11. The case was managed successfully with supportive care and high-dose intravenous immunoglobulins in addition to fluoxetine.

**Discussion and conclusions:**

This patient’s unique presentation and clinical course presents important implications for the care of immunosuppressed patients with cryptic complaints.

## Introduction

Multiple sclerosis (MS) is a chronic, inflammatory demyelinating autoimmune disease that affects the central nervous system, leading to varying degrees of physical and cognitive impairment. Patients with relapsing–remitting MS are often treated with disease-modifying therapies, including anti-CD20 monoclonal antibodies such as ocrelizumab, rituximab, ofatumumab, and ublituximab. These antibodies deplete B cells, which contribute to the pathogenesis of MS through mechanisms such as cytokine regulation, antigen presentation, and autoantibody production [1]. However, their use is also associated with an increased risk of infections (particularly respiratory and skin infections), infusion reactions, and hypogammaglobulinemia. Especially Ocrelizumab carries an additional risk of immune-mediated colitis and breast cancer, and it is contraindicated for patients with active hepatitis B due to the potential for viral reactivation. Here, we report the case of a young female who developed severe disseminated enterovirus infection, complicated by hepatitis, enterocolitis and pancreatitis during the maintenance phase of chemotherapy.

## Case report

**Fig. 1:**
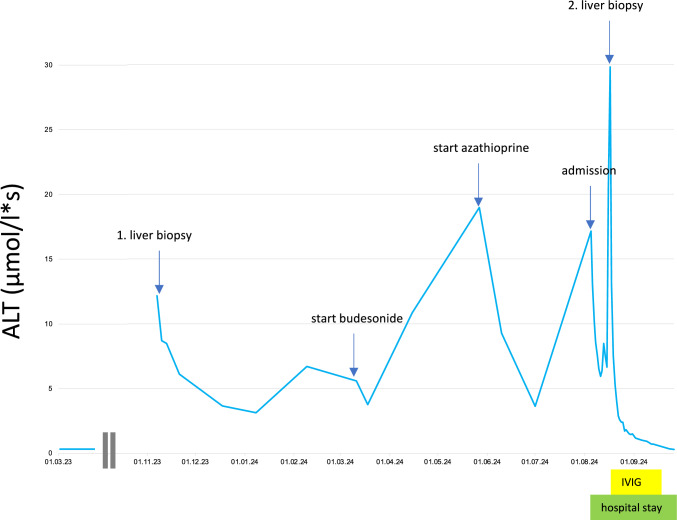
Course of ALT of the patient in 2023 and 2024. Relevant diagnostic and therapeutic events are indicated

We present the case of a 20-year-old female patient previously diagnosed with multiple sclerosis, who was referred to our hospital in August 2024 due to elevated liver enzyme alanine aminotransferase (ALT) and aspartate aminotransferase (AST) levels, along with a decline in her general condition. She had a history of increased liver enzymes and a liver biopsy indicated autoimmune hepatitis (AIH) combined with drug-induced alterations in November 2023. This was initially treated with steroids starting in November 2023, followed by budesonide in March 2024 and azathioprine in May 2024 without achieving a normalization of the liver enzymes (Fig. 1). At admission to the hospital, she used azathioprine 50 mg and budesonide 9 mg daily.

The increase in liver enzymes that led to the current admission was initially suspected to be a flare of the suggested AIH, and as a result, the steroid dose was increased to 50 mg/day. Notably, she had been treated for her MS with ocrelizumab until September 2023, when the therapy was discontinued due to the sudden acute left-sided hearing loss and severe sensorineural hearing loss on the right side. On admission, the ALT was elevated to 13.03 mmol/l*s (normal < 0.58 µmol/l*s) and the AST to 10.41 µmol/l*s (normal < 0.60 µmol/l*s) (Fig. 1), while liver function was impaired with Bilirubin at 86 µmol/l (normal < 21 µmol/l) and Albumin at 26 g/l (normal > 35 µmol/l). Liver synthesis as indicated by Quick was unaffected at admission (96%) but dropped to a minimum of 43% in the course of the disease. Additionally, markers of inflammation were elevated with CRP at 49.8 mg/l. Given her previous treatment with ocrelizumab, flow cytometric was performed revealing a complete depletion of CD19-positive B lymphocytes. Immunoglobulin deficiency was also noted, with a total IgG of 5.1 g/L (normal 7–16 g/l) and a significant IgM deficiency of 0.08 g/L (normal 0.4–2.3 g/l). Both cytomegaly virus PCR and Epstein Barr Virus PCR in the blood were below the limit of detection. Imaging studies indicated gastroenteritis and oedematous pancreatitis. A CT-scan also revealed severe pneumonia. Urine diagnostics, including a urine protein profile, showed severe tubular proteinuria, with a peak level of alpha-1-microglobulin of 698.7 mg/g creatinine. Analysis of throat rinse fluid resulted in a positive detection of enteroviruses with no evidence of SARS-CoV-2. Subsequently, a bronchoscopy with bronchoalveolar lavage (BAL) was performed, which again showed evidence of Enteroviridae. Additionally weakly positive results for *Pneumocystis jirovecii* and *Herpes Simplex Virus I* were identified. No bacterial pathogen could be detected. Shortly after admission, the patient´s condition significantly worsened, marked by a further increase in liver enzymes (AST max 104.41 µmol/l*s). The patient presented with tachycardia (140 bpm), arterial hypotension (systolic blood pressure 90 mmHg), somnolence and respiratory failure requiring intubation and mechanical ventilation, leading to her transfer to the intensive care unit. Clinically relevant ascites also developed. Since the initial imaging and BAL indicated pneumonia, with positive PCR results for *Pneumocystis jiroveci*i and Herpes simplex virus, a therapy with meropenem, cotrimoxazole and aciclovir was initiated despite unclear pathological relevance of the identified pathogens in the current case.

**Fig. 2:**
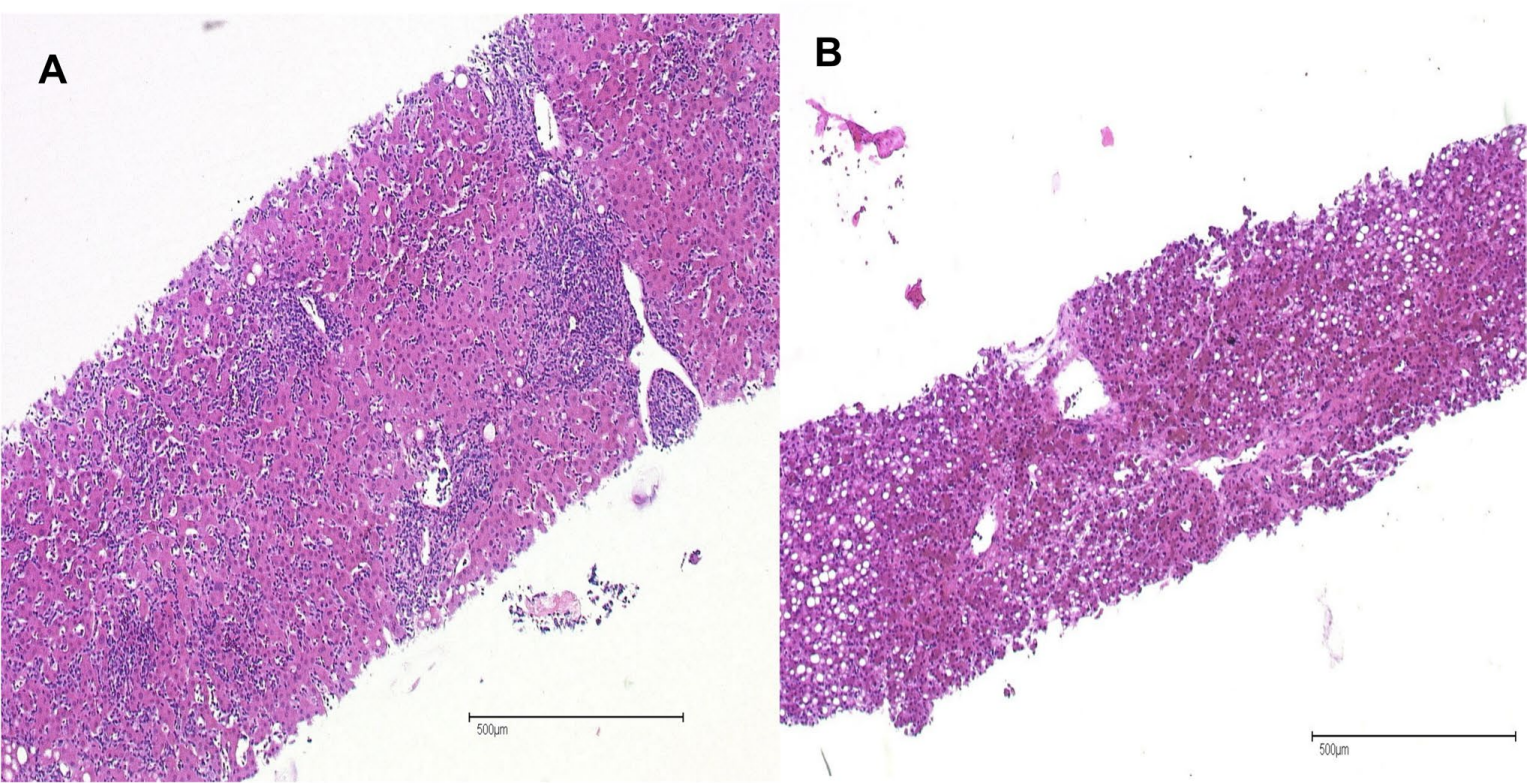
**a** (2023) Liver biopsy with portal and also intraacinar inflammation. Interface hepatitis, perivenular necrosis and mild fibrosis. Rosettes and emeperiplosis is missing. very few fat droplets (Hematoxylin–Eosin). **b** (2024): In the subsequent liver biopsy, the inflammatory cells decreased—and a marked steatosis occured with a diffuse macro-microvesicular pattern (Hematoxylin-Eosin)

In the context of massive increase in liver enzymes, elevated bilirubin, reduced Quick, and suspected AIH from the first biopsy, the somnolence was interpreted as a hepatic encephalopathy raising suspicion of acute liver failure. As the increase in steroid dose failed to improve both clinical and laboratory findings, a second liver biopsy was performed to refine the etiological diagnosis of the hepatitis. The re-biopsy revealed mild portal and intra-acinar inflammation, along with pronounced liver steatosis affecting 70% of the hepatocytes, significant cholestasis, and mild to moderate fibrosis. These findings were interpreted as a hepatic reaction to a prolonged systemic enterovirus infection. In contrast, the previously described histopathological changes indicative of AIH was not observed in the current biopsy (Fig. 2 a and b). Re-imaging additionally showed signs of pleural fluid collections. Both, the ascites and the pleural fluid were punctured and microbiological diagnostics revealed positive results for Enterovirus. A positive enterovirus PCR from plasma was identified, confirming echovirus serotype 11 after serotyping at the national reference centre for Polio- and Enterovirus-infections. At initiation of therapy, the Ct value for Enterovirus spp. was 29.1, increased to 31.8 after six days of therapy and was negative 12 days after therapy initiation. As a limitation, the Ct value was measured with a non-validated method and for clinical decision making, only the information positive or negative was used.

Considering these results, a systemic enterovirus infection with liver involvement was suspected. Intravenous immunoglobulins were administered due to secondary immunodeficiency with accompanying immunoglobulin deficiency. A cumulative dose of 120 g of Octagam^®^ and 40 g of Privigen^®^ was given over a period of three weeks. Based on the previously reported antiviral effect of fluoxetine, adjunctive therapy with 20 mg/day over 8 days, followed by 40 mg/day over 28 more days. Steroids were gradually reduced and stopped after 39 days. Under the chosen therapy, subsequent enterovirus PCR from both, blood and pleural punctate consistently remained below the detection threshold.

The patient´s general condition improved, and she was extubated after 2 days, requiring high-flow oxygen supplementation for an additional 6 days. Her liver enzymes rapidly decreased and returned to normal values. (Fig. 1) Unfortunately, the hearing-loss, which was retrospectively attributed to the enterovirus infection, persisted despite the successful therapy of the systemic infection. After a total length of stay of 53 days, the patient was discharged to a rehabilitation centre.

## Discussion

This case represents a severe systemic enterovirus infection in a young female patient with iatrogenic immunodeficiency induced by ocrelizumab. Retrospective examination revealed that her medical history began with an unexplained bilateral severe hearing loss, followed by hepatitis with an increase in bilirubin levels, a drop in blood pressure and somnolence – meeting the criteria for acute liver failure.

Enteroviruses causing deafness have been reported in the literature, but this constellation is very rare; it is a serious, rare disease [2–4]. In general, enterovirus infections in children are asymptomatic or mild, but can manifest as severe illness, including sepsis, myocarditis, meningitis, and encephalitis [5]. These disease manifestations can be associated with significant morbidity and mortality, particularly in newborns and young children [6, 7]. A recently published study on severe enterovirus infections on hospitalized children in England demonstrated that coxsackie A viruses, coxsackie B viruses and echoviruses accounted for similar proportions of the severe enterovirus infections with sepsis (n = 9), myocarditis (n = 8), meningitis (n = 8) and encephalitis (n = 5) [8]. Verboon-Maciolek et al. reported three infants who developed liver failure after Echovirus 20 infection [9]. In nine of 16 pediatric patients who had an etiologically unclear abnormal liver function, using molecular genetic diagnostics, enteroviruses were detected in the liver [10]. In contrast to infants and young children in whom enterovirus infections are often relatively bland, serious illnesses such as hepatitis rarely occur in adults. These are usually immunodeficient patients [11, 12]. Lefterova et al. reported a case of ALF in an adult caused by echovirus 18 infection. This patient had a history of non-Hodgkin lymphoma and received a hematopoietic stem cell transplant [11]. Additionally, a case of an Echoviral infection in a patient with multiple sclerosis is reported [13].

To the best of our knowledge, there have been no previously reported cases of severe systemic enterovirus-related infections associated with the use of ocrelizumab. Prevalence of hypogammaglobulinemia among MS patients treated with anti-CD20 antibodies is a usual phenomenon. Looking at different subgroups reveals that rituximab has the highest prevalence at 18%, followed by ocrelizumab at 11%, non-specified anti-CD20 at 10%, and ofatumumab at 2%. Chronic usage of anti-CD20 therapies has been associated with a decline in immunoglobulin levels, particularly IgG and IgM. The decrease in IgG levels may persist even after the discontinuation of therapy, as it was the case in our patient. Evidence from certain clinical trials and observational studies has suggested an relationship between Ig antibody levels and infection rates, as well as infection severity in patients with MS [14, 15] However, a large real world cohort study demonstrated that at least one individual hypogammaglobulinemia event occurred in 32.8% of ocrelizumab-treated patients for IgM and 21 serious infections in 266 patients occurred without any association between hypogammaglobulinemia and serious infection risk [16]. If the causal significance of hypogammaglobulinemia is unclear, it is important that the patient was treated with azathioprine and budesonide for presumed autoimmune hepatitis. In 11/2023 there was an increase in transaminases and autoantibody diagnostics could not be used due to B cell depletion. The histology carried out also revealed (clear) evidence of autoimmune hepatitis when viewed retrospectively (Fig. 2a). An infection diagnosis carried out at that time (without a specific search for enteroviruses) produced negative results. Knowing the subsequent course of the patient´s conditions, we retrospectively attempted to identify Enterovirus spp. in the initial liver sample. Unfortunately, we could not detect enteroviral fragments. It is noteworthy, that we had access only to paraffin-fixed material from that sample. Furthermore, it should be emphasized that the routine tests used for detection of Enterovirus spp. are not validated for use of paraffin-fixed samples, which may result in false-negative outcomes. However, from a critical standpoint, systemic involvement of the liver in an already existing enterovirus infection in November 2023 appears unlikely. Under combined azathioprine/budesonide therapy, transaminases failed to normalize and an increase occurred in July 2024 (Fig. 1). The second liver puncture performed at this time revealed a different histological finding (Fig. 2b). In contrast to the initial picture, a predominant steatosis was now observed with a reduced level of inflammation. The PCR for enteroviruses carried out at the same time from the liver was highly positive. Given the virus detection in the liver, blood, BAL, pleural effusion and stool, the diagnosis of enterovirus viremia was made. With supportive intensive care therapy, high-dose immunoglobulin administration and fluoxetine, the patient’s condition was stabilized in line with a reduction of the viral load. While high-dose immunoglobulin administration is an integral part of therapy in case studies of severe enterovirus infections [17, 18], there are only rare data for a rationale to use fluoxetine. Fluoxetine is a selective serotonin reuptake inhibitor commonly used as an antidepressant in psychiatric patients, but there is also evidence of positive effects in enterovirus infections [19–23]. Very recently, Yu et a described a case of autosomal recessive agammaglobulinemia (ARA) featuring a homozygous CD79a mutation long-term chronic enteroviral meningitis was successfully treated using a combination of intravenous immunoglobulin and fluoxetine, further reinforcing fluoxetine’s efficacy in the treatment of enteroviral meningitis [24]. If you look at it critically, the use of fluoxetine is not evidence-based, but it could be an option for refractory disease in these patients, and therefore a higher clinical suspicion for this disease in the setting of the above clinical indicators may have facilitated a more active treatment.

## Conclusion

As ocrelizumab is becoming more widely used to treat MS, healthcare providers must be aware of the potential link between the medication and potentially life-threatening opportunistic infections. Disseminated enterovirus infection can be serious in immunocompromised patients. Our case report is intended to encourage physicians treating immunocompromised patients, especially patients with humoral deficiency, to consider enterovirus infection in cases of unclear neurological, hepatic, gastrointestinal and/or pancreatic diseases. The treatment success in our case by using immunoglobulins and fluoxetine justifies the use of this regimen in similar cases, although controlled studies would also be of great interest.

## Data Availability

Data underlying the MS is available from the corresponding author upon reasonable request.
